# *LogSum* + *L*_2_ penalized logistic regression model for biomarker selection and cancer classification

**DOI:** 10.1038/s41598-020-79028-0

**Published:** 2020-12-17

**Authors:** Xiao-Ying Liu, Sheng-Bing Wu, Wen-Quan Zeng, Zhan-Jiang Yuan, Hong-Bo Xu

**Affiliations:** Computer Engineering Technical College, Guangdong Polytechnic of Science and Technology, Zhuhai, 519090 Guangdong China

**Keywords:** Computational biology and bioinformatics, Mathematics and computing

## Abstract

Biomarker selection and cancer classification play an important role in knowledge discovery using genomic data. Successful identification of gene biomarkers and biological pathways can significantly improve the accuracy of diagnosis and help machine learning models have better performance on classification of different types of cancer. In this paper, we proposed a *LogSum* + *L*_2_ penalized logistic regression model, and furthermore used a coordinate decent algorithm to solve it. The results of simulations and real experiments indicate that the proposed method is highly competitive among several state-of-the-art methods. Our proposed model achieves the excellent performance in group feature selection and classification problems.

## Introduction

With the development of DNA microarray technology^1,2^, the biological researchers can analyze the expression levels of thousands of genes simultaneously. Many studies have shown that microarray data can be used to classify the different types of cancer, which includes how long the incubation period is, and what drugs are effective in the diagnosis and treatment processes.

From a biological point of view^3^, only a small number of genes (biomarkers) strongly indicate the target cancer, while other genes are not related to disease. Therefore, the data with unrelated genes may bring noise, and make the machine learning approaches less easy to find pathogenic genes that cause the disease. Moreover, from a machine learning perspective, the large number of genes (features) with few samples in the datasets may cause overfitting^4^, and have negative impact on classification performance. Due to the importance of these issues, effective gene (biomarker) selection methods are needed to help classify different cancer types and improve prediction accuracy.

In recent years, many methods for gene selection in microarray datasets have been developed and generally can be divided into three categories: filters, wrappers, and embedded methods. Filter methods^5–8^ evaluate genes based on discriminative power without considering their regulation correlations with other genes. The main disadvantage of the filtering methods is that it examines each gene separately, and makes each gene independent, thereby ignores the possibility that the genes have combined and grouping effects. This is a common problem with statistical methods, such as *t*-test, which can also examine each gene individually.

Wrapper methods^9–11^ utilize feature assessment measures based on the learning performance to select subsets of genes. Generally, they can acquire a small number of related genes to notable promote the learning ability. In some cases, the results of the wrapper methods are better than those of the filter methods. However, the main fault of wrapper methods is their computational cost is high.

A third set of feature selection approaches is the embedded methods^12–26^ that perform feature selection as part of the learning procedure of a single process. Under similar learning performance, the computational efficiency of embedded methods is more efficient than wrapper approaches. Hence, embedded methods have recently attracted a lot of attention in the literature. The regularization methods are important embedded technologies, which can perform feature selection and model training simultaneously. Many regularization methods have been proposed, such as Lasso^12^, SCAD^13^, adaptive Lasso^14^, MCP^15^, *L*_q_ (0 < *q* < 1)^16^, *L*_1/2_^17,18^, *LogSum*^19^, etc. These methods perform well with the independent feature selection. When the features are highly correlated, some regularization methods which pay attention to the grouping effect can be used to select the groups of the relevant features, such as group Lasso^20^, Elastic net^21^, Fused Lasso^22^, OSCAR^23^, adaptive Elastic net^2,4^, SCAD-*L*_2_^2,5^, *L*_1/2_ + *L*_2_^2,6^.

On the other hand, many machine learning models have been used to analyze microarray gene expression data for cancer classification. For example, Furey et al. used support vector machines (SVMs) to classify cell and tissue types^27^. Medjahed et al. applied the K-nearest neighbors (K-NN) to the diagnosis and classification of breast cancer^28^. Meanwhile, some researchers used the logistic regressions with optimization methods for binary cancer classification^29–33^. However, the traditional logistic regression model has two obvious shortcomings, mainly in the following two aspects:

1. Feature selection problem.
  All or most of the feature coefficients obtained by fitting the logistic regression model are not zero, i.e. all most of the features are related to the classification target and not sparse. However, the key factors affecting the model are often only a few in many practical problems. This non-sparseness of the logistic models increases the computational complexity on the one hand and is not conducive to the actual interpretation of the practical problems.
2. Overfitting problem.
  The logistic regression models can often obtain good precision for the training data, but for the test data outside the training set, the classification accuracy rate is not ideal. In fact, not only logistic regression, many other data analysis models will also be affected by overfitting. It has become one of the hot research topics in statistics, machine learning and other fields.

In recent years, there is growing interesting to apply the regularization techniques in the logistic regression models to solve the above mentioned two shortcomings. For example, Tibshirani and Friedman^34,35^ proposed the sparse logistic regression based on the Lasso regularization and the coordinate descent methods. Algamal et al.^36,37^ proposed the adaptive Lasso and the adjusted adaptive elastic net for gene selection in high dimensional cancer classification. Like sparse logistic regression with the *L*_1_ regularization method, Cawley and Talbot^30^ investigated sparse logistic regression with Bayesian regularization. Liang et al.^3,8^ investigated the sparse logistic regression model with the *L*_1/2_ penalty for gene selection in cancer classification.

Inspired by above mentioned methods, in this paper, we proposed a *LogSum* + *L*_2_ penalized logistic regression model. The main contributions of this paper include.

1. Our proposed method can not only select sparse features (biomakers), but also identify the groups of the relevant features (gene pathways). The coordinate decent algorithm is used to solve the *LogSum* + *L*_2_ penalized logistic regression model.
2. We also evaluate the capability of our proposed method and compare its performance with other regularization methods. The results of simulations and real experiments indicate that the proposed method is highly competitive among several state-of-the-art methods.

The rest of this paper is organized as follows. In “Related works” section, we introduce the related work. “Methods” section represents the *LogSum* + *L*_2_ penalized logistic regression model and its optimization algorithm. “Experiments experimental results and discussion” section analyzes the results of the simulated data. “Discussion and conclusion” section analyzes the results of real data. Section 6 concludes this paper.

## Related works

### Sparse penalized logistic regression

We focused on binary classification using logistic regression (*LR*), which is a statistical method for modeling a binary classification problem. Suppose we have *n* samples and *p* genes. Datasets *X* and *y* are the genes matrix and the dependent variable, respectively. So, the *n* samples mean the set *D*, $x_{ij}$ denotes the value of gene $j$ for the $i{\rm th}$ samples, *y*_*i*_ is a corresponding variable that takes a value of 0 or 1, $y_{i}$ = 0 indicates the $i{\rm th}$ sample in Class 1 and $y_{i}$ = 1 indicates the $i{\rm th}$ sample is in Class 2. Then, we define a classifier $f(x) = \frac{{e^{x} }}{{(1 + e^{x} )}}$ such that for any input *x* with class label $y$,$f(x)$ predicts *y* correctly. The $LR$ is given as follows:

1 $$P(y_{i} = 1|X_{i} ) = f(X_{i}^{\prime } \beta ) = \frac{{{\text{e}}^{{(X_{i}^{\prime } \beta )}} }}{{1 + e^{{(X_{i}^{\prime } \beta )}} }}$$

In Eq. (1), $\beta { = (}\beta_{0} {,}\beta_{1} {,}...{,}\beta_{p} {)}$ are the coefficients need to be estimated. We should notice that $\beta_{0}$ is the intercept. The log-likelihood function of the transformation of Eq. (1) is defined as:

2 $$l(\beta ) = - \sum_{i = 1}^{n} {\{ y_{i} \log [f(X_{i}^{\prime } \beta )] + (1 - y_{i} )\log [1 - f(X_{i}^{\prime } \beta )]\} }$$

Then we can obtain the coefficients $\beta$ when Eq. (2) is minimized. In the cancer classification problem with high-dimensional and low-sample size data $(p \gg n),$directly solving the logistic model (2) will make overfitting. Therefore, to solve this problem, we need add a regularization term to (2), the sparse logistic regression can be modelled as:

3 $$\beta = argmin\left\{ l(\beta ) + \lambda \sum_{j = 1}^{p} {p(\beta_{j} )} \right\}$$

where $l(\beta )$ is the loss function, $p(\beta )$ is the penalty function, and $\lambda > 0$ is a control parameter.

### A coordinate decent algorithm for different thresholding operators

The coordinate decent algorithm is a “one-at-a-time” approach^40^, and before considering the coordinate descent algorithm for the nonlinear logistic regularization, we first introduce a linear regression case. The objective function of the linear regression is as follow:

4 $$min\left\{ {\frac{1}{2n}|| y - X\beta||^{2} + P_{\lambda } \left( \beta \right)} \right\}$$

where $y = (y_{1} , \ldots ,y_{n} )^{T}$ is the vector of *n* response variables,$X_{i} = (x_{i1} ,x_{i2} , \ldots ,x_{ij} )$ is *i*th input variables with dimensionality $p$ and $y_{i}$ is the corresponding response variable. $||.||$ denotes the $L_{2}$-norm.

The coordinate decent algorithm “one-at-a-time” is to solve $\beta_{j}$ and other $\beta_{k \ne j}$(represent the coefficients $\beta_{k \ne j}$ remained after $j\hbox{th}$ element $\beta_{j}$ is removed) are fixed. The Eq. (4) can be rewritten as:

5 $$R(\beta ) = argmin\left\{ {\frac{1}{2n}\left( {y_{i} - \left( {\sum_{k \ne j} {x_{ik} \beta_{k} + x_{ij} \beta_{j} } } \right)} \right)^{2} + \lambda \sum_{k \ne j} {P(\beta_{k} ) + } \lambda P(\beta_{j} )} \right\}$$

In Eq. (5), *k*th represents other features than the *j*th feature.

The first order derivative at $\beta_{j}$ can be estimated as:

6 $$\frac{\partial R}{{\partial \beta_{j} }} = \sum_{i = 1}^{n} {\left( { - x_{ij} \left( {y_{j} - \sum_{k \ne j} {x_{ik} \beta_{k} - x_{ij} \beta_{j} } } \right)} \right) + \lambda P(\beta_{j} ) = 0}$$

We define $\tilde{y}_{i}^{\left( j \right)} = \mathop \sum _{k \ne j} x_{ik} \beta_{k}$ as a part of fitting $\beta_{j}$, $\tilde{r}_{i}^{\left( j \right)} = y_{i} - \tilde{y}_{i}^{\left( j \right)}$, and $w_{j} = \sum_{i = 1}^{n} {x_{ij} \tilde{r}_{i}^{(j)} }$, where $\tilde{r}_{i}^{(j)}$ represents the partial residuals with respect to the *j*th feature.

To consider the correlation of features, Elastic Net ($L_{EN}$)^21^ had been proposed, which emphasizes a grouping effect. The $L_{EN}$ penalty function is given as follows:

7 $$P(\beta ) = (1 - a)\frac{1}{2}||\beta||_{{L_{2} }}^{2} + a||\beta||_{{L_{1} }}$$

The penalty function of $L_{EN}$ is combination of $L_{1}$ penalty and ridge penalty which $a = 1$ and $a = 0$ respectively. Therefore, Eq. (6) is rewritten as follows:

8 $$\frac{\partial R}{{\partial \beta_{j} }} = \sum_{i = 1}^{n} {\left( { - x_{ij} \left( {y_{j} - \sum_{k \ne j} {x_{ik} \beta_{k} - x_{ij} \beta_{j} } } \right)} \right) + \lambda (1 - a)\beta_{j} + \lambda a = 0}$$

Zou and Hastie have proposed the univariate solution^21^ for a $L_{EN}$ penalized regression coefficient as follows:

9 $$\beta_{j} = f_{{L_{EN} }} (w_{j} ,\lambda ,a) = \frac{{S(w_{j} ,\lambda a)}}{1 + \lambda (1 - a)}$$

where $S(w_{j} ,\lambda a)$ is soft thresholding operator for the $L_{1}$ penalty if *a* is equal to 1, so Eq. (9) can be divided into three situations as follows:

10 $$\beta_{j} = Soft(w_{j} ,\lambda ) = \left\{ {\begin{array}{*{20}l} {w_{j} + \lambda } \hfill & {\quad {\text{if}}\,\, \, w_{j} < - \lambda } \hfill \\ {w_{j} - \lambda } \hfill & {\quad {\text{if}}\,\, \, w_{j} > \lambda } \hfill \\ 0 \hfill & {\quad {\text{if}}\,\, \, - \lambda \le w_{j} \le \lambda } \hfill \\ \end{array} } \right.$$

Fan et al. have proposed the SCAD penalty^13^, which can produce sparse set of solutions and approximately unbiased coefficients for large coefficients. Its penalty function is shown as follows:

11 $$p_{\lambda ,a} (\beta ) = \left\{ {\begin{array}{*{20}l} {\lambda \beta } \hfill & {\quad {\text{if}}\;\; \, \beta \ne \lambda } \hfill \\ {\frac{{a\lambda \beta - \frac{1}{2}(\beta^{2} + \lambda^{2} )}}{a - 1} \, } \hfill & {\quad {\text{if }}\;\;\lambda < \beta < a\lambda \, } \hfill \\ {\frac{{\lambda (a^{2} - 1)}}{2(a - 1)}} \hfill & {\quad {\text{if }}\;\;\beta > a\lambda \, } \hfill \\ \end{array} } \right.$$

Additionally, the SCAD thresholding operator is given as follows:

12 $$\beta_{j} = f_{SCAD} (w_{j} ,\lambda ,a) = \left\{ {\begin{array}{*{20}l} {S(w_{j} ,\lambda )} \hfill & {\quad {\text{if }}\,\,\left| {w_{j} } \right| < 2\lambda } \hfill \\ {\frac{{S\left( {w_{j} ,\frac{a\lambda }{{a - 1}}} \right)}}{{1 - \frac{1}{a - 1}}}} \hfill & {\quad {\text{if}}\;\; \, 2\lambda < \left| {w_{j} } \right| \le a\lambda } \hfill \\ {w_{j} } \hfill & {\quad {\text{if }}\;\;\left| {w_{j} } \right| > a\lambda } \hfill \\ \end{array} } \right.$$

Like the SCAD penalty, Zhang et al. have proposed the maximum concave penalty (MCP)^15^. The formula of its penalty function is shown as:

13 $$p_{\lambda ,a} (\beta ) = \left\{ {\begin{array}{*{20}l} {\lambda \beta } \hfill & {\quad {\text{if}}\;\; \, \beta \le \gamma \lambda } \hfill \\ {\frac{1}{2}\gamma \lambda^{2} } \hfill & {\quad {\text{if }}\;\;\beta > \gamma \lambda } \hfill \\ \end{array} } \right.$$

And the MCP thresholding operator is given as follows:

14 $$\beta_{j} = f_{MCP} (w_{j} ,\lambda ,\gamma ) = \left\{ \begin{array}{*{20}l} \frac{{S(w_{j} ,\lambda )}}{{1 - \frac{1}{\lambda }}} & {\text{if }}\left| {w_{j} } \right| \le \gamma \lambda \hfill \\ w_{j} & {\text{if }}\left| {w_{j} } \right| > \gamma \lambda \hfill \\ \end{array} \right.$$

In Eq. (14), $\gamma$ is the experience parameter.

Xu et al. have proposed $L_{1/2}$ regularization^17^, and its penalty function can be written:

15 $$min\left\{ {\frac{1}{2n}\left\| {y - X\beta } \right\|^{2} + \lambda \sum_{j}^{p} {\left| {\beta_{j} } \right|^{\frac{1}{2}} } } \right\}$$

Then the univariate half thresholding operator for a $L_{1/2}$ penalized linear regression coefficient is given as follows:

16 $$\beta_{j} = Half(w_{j} ,\lambda ) = \left\{ {\begin{array}{*{20}l} {\frac{2}{3}w_{j} \left( {1 + \cos \frac{{2(\pi - \phi_{\lambda } (w_{j} ))}}{3}} \right)} \hfill & {\quad {\text{if}}\;\; \, \left| {w_{j} } \right| > \frac{3}{4}(\lambda )^{\frac{2}{3}} } \hfill \\ 0 \hfill & {\quad {\text{if }}\;\;otherwise} \hfill \\ \end{array} } \right.$$

in Eq. (16), $$\phi_{\lambda } (w) = \frac{\lambda }{8}\left( {\frac{\left| w \right|}{3}} \right)^{{ - \frac{3}{2}}}$$.

To consider the correlation of genes, Huang et al. have proposed $HLR$ regularization^26^. Equation (15) can be rewritten:

17 $$min\left\{ {\frac{1}{2n}\left\| {y - X\beta } \right\|^{2} + \lambda \left( {\sum_{j}^{p} {\left( {a\left| {\beta_{j} } \right|^{\frac{1}{2}} + (1 - a)\left| {\beta_{j} } \right|^{2} } \right)} } \right)} \right\}$$

And the univariate half thresholding operator for the $HLR$ penalized linear regression coefficient is as follows:

18 $$\beta_{j} = HLR(w_{j} ,\lambda ) = \frac{{Half(w_{j} ,\lambda a)}}{1 + \lambda (1 - a)}$$

Theoretically, the *L*_0_ regularization produces the better solutions with more sparsity, but it is *NP* problem. Therefore, Candes et al. have^19^ proposed $LogSum$ penalty, which approximates much better the $L_{0}$ regularization. We could rewrite the penalty function of the $LogSum$ regularization as follows:

19 $$min\left\{ {\frac{1}{2n}\left\| {y - X\beta } \right\|^{2} + \lambda \sum_{j}^{p} {log(\left| {\beta_{j} } \right| + \varepsilon )} } \right\}$$

where $\varepsilon > 0$ should be set arbitrarily small, to closely make the $LogSum$ penalty resemble the *L*_0_-norm. Equation (19) has a local minimal^3,9^.

20 $$f_{Logsum} (w_{j} ,\lambda ,\varepsilon ) = D(w_{j} ,\lambda ,\varepsilon ) = \left\{ {\begin{array}{*{20}l} {sign(w_{j} )\frac{{c_{1} + \sqrt {c_{2} } }}{2}} \hfill & {\quad {\text{if}}\;\; \, c_{2} > 0} \hfill \\ 0 \hfill & {\quad {\text{if}}\;\; \, c_{2} \le 0} \hfill \\ \end{array} } \right.$$

where $$\lambda > 0$$, $$0 < \varepsilon < \sqrt \lambda$$, $$c_{1} = w_{j} - \varepsilon$$, $$c_{2} = c_{1}^{2} - 4(\lambda - w_{j} \varepsilon )$$.

## Methods

### ***LogSum*** + ***L***_2_ penalized logistic regression model

In this paper, we proposed the *LogSum* + *L*_2_ penalized logistic regression model for feature group selection. We could write the *LogSum* + *L*_2_ penalty as follows:

21 $$min\left\{ {\frac{1}{2n}\left\| {y - X\beta } \right\|^{2} + \lambda \left( {\sum_{j}^{p} {\left( {\lambda_{1} log\left( {\left| {\beta_{j} } \right| + \varepsilon } \right) + \lambda_{2} \left| {\beta_{j} } \right|^{2} } \right)} } \right)} \right\}$$

where $\left\| {y - X\beta } \right\|^{2}$ is the loss function, $(y,X)$ is a data set, $\varepsilon > 0$ is a constant, $\lambda>0$, $\lambda_{1} \ge 0$ and $\lambda_{2} \ge 0$ are regularization parameters that control the complexity of the penalty function.

**Figure 1:**
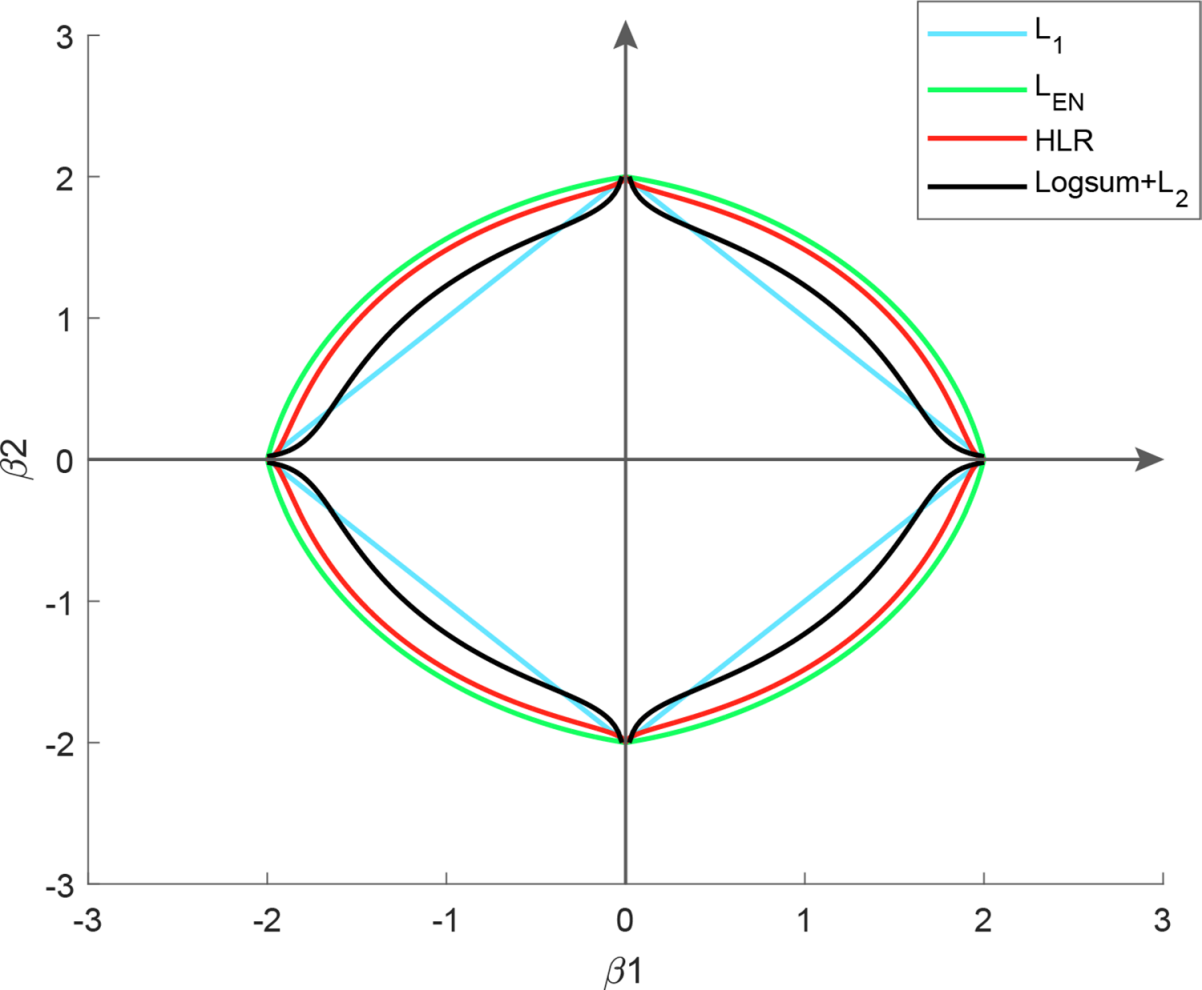
Contour plots (two-dimensional) for the regularization methods.

Figure 1 describes the contour plots on two-dimensional for the penalty functions of *L*_1_, *L*_*EN*_, *HLR* and *LogSum* + *L*_2_ approaches. It is demonstrated that the *LogSum* + *L*_2_ penalty is non-convex for the given parameters ${\lambda }_{1}$ and ${\lambda }_{2}$ in Eq. (21).

The *LogSum* + *L*_2_ thresholding operator is given as follows:

22 $${\beta }_{j}={f}_{Logsum+{L}_{2}}\left({w}_{j},\lambda ,\varepsilon \right)=\left\{\begin{array}{ll}sign\left({w}_{j}\right)\frac{\left(\left|{w}_{j}\right|-\left(1+2{\lambda }_{2}\right)\varepsilon \right)+\sqrt{{\left(\left|{w}_{j}\right|+\left(1+2{\lambda }_{2}\right)\varepsilon \right)}^{2}-4{\lambda }_{1}\left(1+2{\lambda }_{2}\right)}}{2\left(1+2{\lambda }_{2}\right)}& if\,\, \left|{w}_{j}\right|>\lambda \\ 0 & if\,\, otherwise\end{array}\right.$$

where $${\lambda} = 2\sqrt {\lambda_{1} \left( {1 + 2\lambda_{2} } \right)} - \left( {1 + 2\lambda_{2} } \right)\varepsilon$$, $$\lambda_{1} + \lambda_{2} = 1$$.

The proof of Eq. (22) is given as follows:

Considering the regression model has the following form

23 $$y = X\beta + e$$

where the response $y \in R^{n}$ , the predictors $X = (x_{1} ,x_{2} ,...,x_{p} ),X \in R^{n \times p}$ and the error term $e = (e_{1} ,e_{2} ,...,e_{n} )$ are i.i.d. with mean 0 variance $\sigma^{2}$.

The $Logsum + L_{2}$ regularization can be expressed as:

24 $$l_{{Logsum + L_{2} }} (\beta ;\lambda_{1} ,\lambda_{2} ) = \frac{1}{2}\left\| {y - X\beta } \right\|^{2} + \sum_{j = 1}^{p} {(\lambda_{1} \log \left(\left| {\beta_{j} + \varepsilon } \right|\right) + \lambda_{2} \beta_{j}^{2} )}$$

Its first partial derivative with respect to $\beta_{k}$ is given by follows:

25 $$\begin{aligned} \frac{{\partial l_{{Logsum + L_{2} }} }}{{\partial \beta_{k} }} & = \sum_{i = 1}^{n} {\left( {y_{i} - \sum_{j = 1}^{p} {x_{ij} \beta_{j} } } \right)( - x_{ik} )} + \frac{{\lambda_{1} {\text{s}} ign(\beta_{k} )}}{{\left| {\beta_{k} } \right| + \varepsilon }} + 2\lambda_{2} \beta_{k} \\ & = \sum_{i = 1}^{n} {\left( {y_{i} - \sum_{j \ne k}^{p} {x_{ij} \beta_{j} } - x_{ik} \beta_{k} } \right)( - x_{ik} )} + \frac{{\lambda_{1} {\text{s}} ign(\beta_{k} )}}{{\left| {\beta_{k} } \right| + \varepsilon }} + 2\lambda_{2} \beta_{k} \\ & = \sum_{i = 1}^{n} {\left( {y_{i} - \sum_{j \ne k}^{p} {x_{ij} \beta_{j} } } \right)( - x_{ik} )} + \sum_{i = 1}^{n} {x_{ik}^{2} \beta_{k} + } \frac{{\lambda_{1} {\text{s}} ign(\beta_{k} )}}{{\left| {\beta_{k} } \right| + \varepsilon }} + 2\lambda_{2} \beta_{k} \\ & = \sum_{i = 1}^{n} {\left( {y_{i} - \sum_{j = 1}^{p} {x_{ij} \beta_{j} } } \right)( - x_{ik} )} + \beta_{k} + \frac{{\lambda_{1} {\text{s}} ign(\beta_{k} )}}{{\left| {\beta_{k} } \right| + \varepsilon }} + 2\lambda_{2} \beta_{k} \\ \end{aligned}$$

Equation (25) is obtained from condition that the design matrix $X$ is orthonormal. By setting the first partial derivative equal to zero, we obtain the estimator with its *k*th element $\hat{\beta }_{k}$.

We first considers the situation $\beta_{j} > 0$, let $r_{i}^{(k)} = y_{i} - \sum_{j \ne k}^{p} {x_{ij} \beta_{j} }$, ${w}_{k}=\sum_{i=1}^{n}{r}_{i}^{\left(k\right)}(-{x}_{ik})$. Set the first partial derivative $\frac{{\partial l_{{Logsum + L_{2} }} }}{{\partial \beta_{k} }} = 0$, we have:

26 $$-{w}_{k}+{\beta }_{k}+\frac{{\lambda }_{1}}{{\beta }_{k}+\varepsilon }+2{\lambda }_{2}{\beta }_{k}=0$$

and Eq. (26) is equivalent to follows:

27 $$\left(1+2{\lambda }_{2}\right){\beta }_{k}^{2}-\left({w}_{k}-\left(1+2{\lambda }_{2}\right)\varepsilon \right){\beta }_{k}-{w}_{k}\varepsilon +{\lambda }_{1}=0$$

Let

$$\begin{aligned}\Delta &={\left({w}_{k}-\left(1+2{\lambda }_{2}\right)\varepsilon \right)}^{2}-4\left(1+2{\lambda }_{2}\right)\left({\lambda }_{1}-{w}_{k}\varepsilon \right) \\ &={\left({w}_{k}+\left(1+2{\lambda }_{2}\right)\varepsilon \right)}^{2}-4{\lambda }_{1}\left(1+2{\lambda }_{2}\right) \end{aligned}$$

We discuss the solutions of Eq. (27) according to the value of $\Delta$.

1. if $\Delta < 0$, Eq. (27) has no solution, that is no real root.
2. if $\Delta = 0$, Eq. (27) has unique root, that is ${\widehat{\beta }}_{k}=\frac{{w}_{k}-\left(1+2{\lambda }_{2}\right)\varepsilon }{2\left(1+2{\lambda }_{2}\right)}$.
3. if $\Delta > 0$, Eq. (27) has two roots, we have
  $${{(w}_{k}+\left(1+2{\lambda }_{2}\right)\varepsilon )}^{2}>4{\lambda }_{1}\left(1+2{\lambda }_{2}\right)$$
  $${w}_{k}+\left(1+2{\lambda }_{2}\right)\varepsilon >2\sqrt{{\lambda }_{1}\left(1+2{\lambda }_{2}\right)}$$

Therefore, when ${w}_{k}\ge 2\sqrt{{\lambda }_{1}\left(1+2{\lambda }_{2}\right)}-\left(1+2{\lambda }_{2}\right)\varepsilon$, we obtain the estimator

28 $${\widehat{\beta }}_{k}=\frac{{w}_{k}-\left(1+2{\lambda }_{2}\right)\varepsilon +\sqrt{{\left({w}_{k}+\left(1+2{\lambda }_{2}\right)\varepsilon \right)}^{2}-4{\lambda }_{1}\left(1+2{\lambda }_{2}\right)}}{2\left(1+2{\lambda }_{2}\right)}$$

For ${\beta }_{k}<0$, we can obtain the estimator in a similar way. Finally, we obtain the thresholding function of the $Logsum + L_{2}$ regularization as Eq. (22).

**Figure 2:**
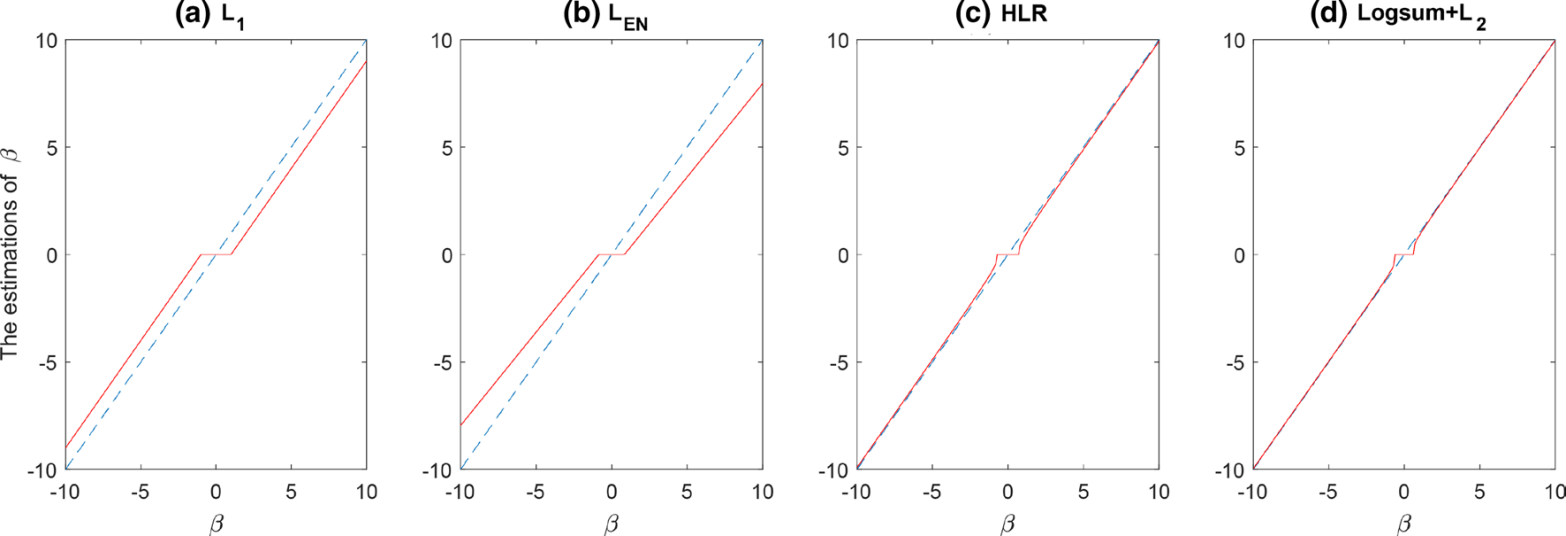
Exact solution of (**a**) $L_{1}$ (**b**) $L_{EN}$ (**c**) $HLR$ (**d**) $LogSum + L_{2}$ in an orthogonal design.

According to different thresholding operators, we also discuss three properties to satisfy the coefficient estimator as shown in Fig. 2:

- *Unbiasedness* the resulting estimator is nearly unbiased when the true unknown parameter is large to avoid unnecessary modeling bias;
- *Sparsity* the resulting estimator is a thresholding rule, which automatically sets a small estimated coefficient to zero to reduce model complexity;
- *Continuity* the resulting estimator is continuous to avoid instability in model prediction.

**Figure 3:**
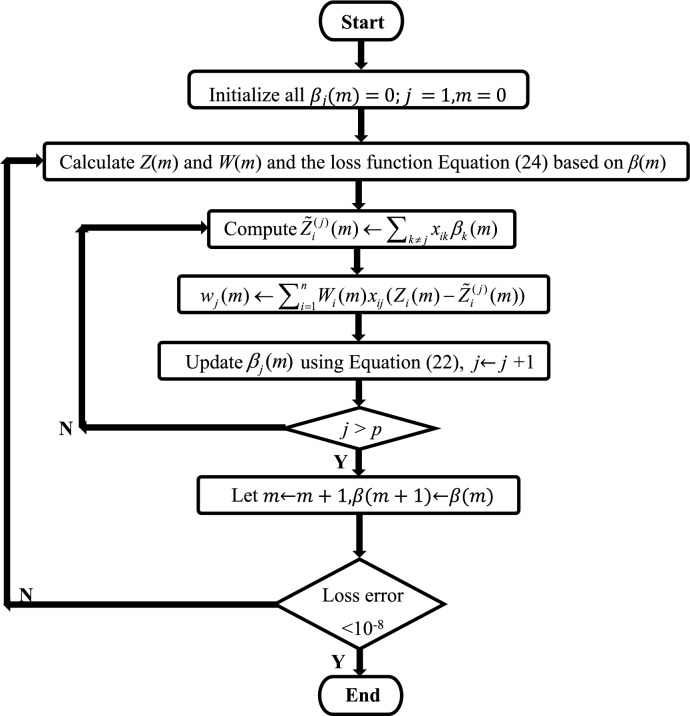
Flowchart of the coordinate descent algorithm for the $LogSum + L_{2}$ penalized logistic regression model.

Figure 2 shows four regularization methods:$L_{1}$, $L_{EN}$, $HLR$ and $LogSum + L_{2}$ penalties with an orthogonal design matrix in the regression model. The estimators of $L_{1}$ and $L_{EN}$ are biased, whereas the $HLR$ penalty is asymptotically unbiased. Similar to the $HLR$ method, the $LogSum + L_{2}$ approach also performs better than $L_{1}$ and $L_{EN}$ in the property of unbiasedness. All of these four regularization methods fulfil requirements of sparsity and continuity.

### A coordinate decent algorithm for the ***LogSum*** + ***L***_2_ model

Inspired by Liang et al.^3,8^, Eq. (3) is linearized by one-term Taylor series expansion:

30 $$L(\beta ,\lambda ) \approx \left\{ {\frac{1}{2n}\sum_{i = 1}^{n} {(Z_{i} - X_{i} \beta )}^{\prime } W_{i} (Z_{i} - X_{i} \beta ) + \lambda \left( {\sum_{j}^{p} {\left( {\lambda_{1} log\left( {\left| {\beta_{j} } \right| + } \right) + \lambda_{2} \left| {\beta_{j} } \right|^{2} } \right)} } \right)} \right\}$$

where $\varepsilon > 0$, $Z_{i} = X_{i} \tilde{\beta } + \frac{{Y_{i} - f(X_{i} \tilde{\beta })}}{{f(X_{i} \tilde{\beta })(1 - f(X_{i} \tilde{\beta }))}}$ is the estimated response, $W_{i} = f(X_{i} \tilde{\beta })(1 - f(X_{i} \tilde{\beta }))$ is the weight and $f(X_{i} \tilde{\beta }) = \frac{{exp(X_{i} \tilde{\beta })}}{{1 + exp(X_{i} \tilde{\beta })}}$. Redefine the partial residual for fitting current $\tilde{\beta }_{j}$ as $\tilde{Z}_{i}^{(j)} = \sum_{k \ne j} {x_{ik} \tilde{\beta }_{k} }$ and $w_{j} = \sum_{i = 1}^{n} {W_{i} x_{ij} } (Z_{i} - \tilde{Z}_{i}^{(j)} )$. A pseudocode of the coordinate descent algorithm for the $Logsum + L_{2}$ penalized logistic regression model is shown in Algorithm 1 (Fig. 3).
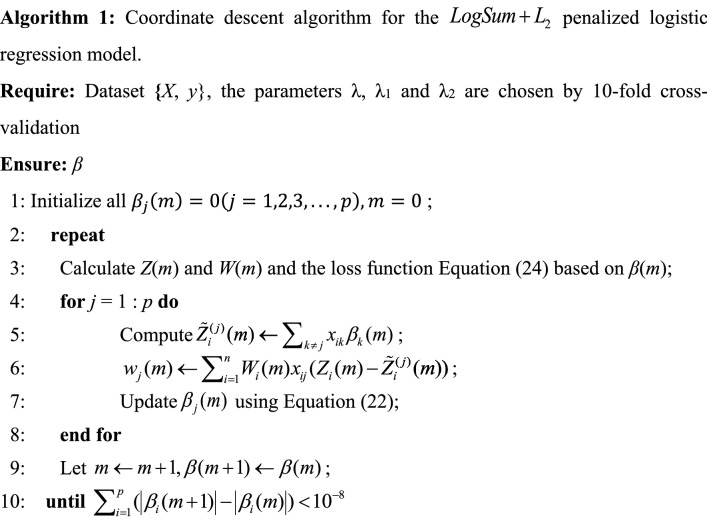


## Experiments experimental results and discussion

### Analysis on simulated data

In this section, we analyze the performance of the proposed method (the $LogSum + L_{2}$ penalized logistic regression model) by simulation analysis. We compare the proposed method with other three methods, which are logistic regression with $L_{1}$, $L_{EN}$, $HLR$ regularizations. We simulate data from the true model.

$$\log \left( {\frac{y}{1 - y}} \right) = X\beta + \sigma \varepsilon , \, \varepsilon \sim N(0, \, 1)$$

where $X\sim N(0, \, 1)$, $\varepsilon$ is the independent random error and σ is the parameter that controls the signal to noise. Two scenarios are presented here. In each example, the dimension of features is 1000. Here are the details of the two scenarios.

1. In Scenario 1, the dataset consists of 200 observations, we set σ = 0.3 and simulate the group feature situation.
  $$\beta = \left( {\underbrace {2,2,2,2,2}_{5},\underbrace {0, \ldots ,0}_{995}} \right);$$
  $$x_{i} = \rho \times x_{1} + (1 - \rho ) \times x_{i} , \, i = 2,3,4,5;$$
  where $\rho$ is the correlation coefficient of the group features.
  In this example, there is one set of related features. The ideal sparse regression method should select 5 real features and set other 995 features as noise features to zero.
2. In Scenario 2, we set σ = 0.4 and the dataset consists of 400 observations, and defined two group features.
  $$\beta = \left( {\underbrace {2,2,2,2,2,1.5, - 2,1.7,3, - 2.5,}_{10}\underbrace {3, \ldots ,3}_{10}\underbrace {0, \ldots ,0}_{980}} \right);$$
  $${x}_{i}=\rho \times {x}_{1}+\left(1-\rho \right)\times {x}_{i},i=\mathrm{2,3},\dots ,10;$$
  $${x}_{i}=\rho \times {x}_{11}+\left(1-\rho \right)\times {x}_{i},i=\mathrm{12,13},\dots ,20;$$

In this example, there are two sets of related group features. The ideal penalized logistic regression method should select 20 real features and set other 980 features as noise features to zero.

In this experiment, we initialize the coefficient $\rho$ of features’ correlation as 0.2, 0.6 respectively, and hope to observe the accuracy of testing under different correlations by running different correlation values. The $L_{1}$ and $L_{EN}$ approaches were executed by Glmnet (http://web.stanford.edu/~hastie/glmnet_matlab/, MATLAB version 2014-a). We use the tenfold cross-validation (CV) approach to optimize the regularization parameters or tuning parameters (balance the tradeoff between data fit and model complexity) of the $L_{1}$, $L_{EN}$, $HLR$ and $LogSum + L_{2}$ approaches.

At the beginning, we divided the datasets at random into the training sets and the test sets. In our experiment, the approximate 70% of samples are proposed as training sets, and the rest are used as test sets. We repeated the simulations 30 times for each penalty method and computed the mean classification accuracy, mean classification sensitivity, and mean classification specificity on the training and test datasets respectively. To evaluate the quality of the selected features for the regularization approaches, the sensitivity and specificity of the feature selection performance^39^ were defined as the follows:

$${\text{True}}\;{\text{Negative}}\;\;(TN): = \left| {\overline{\beta }.*\overline{\hat{\beta }}} \right|_{0} ,\quad {\text{False }}\;{\text{Positive}}\;\;(FP): = \left| {\overline{\beta }.*\hat{\beta }} \right|_{0}$$

$${\text{False}}\;{\text{ Negative}}\;\;(FN): = \left| {\beta .*\overline{\hat{\beta }}} \right|_{0} ,\quad {\text{True }}\;{\text{Positive}}\;\;(TP): = \left| {\beta .*\hat{\beta }} \right|_{0}$$

$$\beta{\text{-Sensitivity}}: = \frac{TP}{{TP + FN}},\quad \beta{\text{-Specificity}}: = \frac{TN}{{TN + FP}}$$

where the $.*$ is the element-wise product, and $\left| . \right|_{0}$ calculates the number of non-zero elements in a vector, $\overline{\beta }$ and $\overline{\hat{\beta }}$ are the logical “not” operators on the vector $\beta$ and $\hat{\beta }$.

The training results of different methods on simulate datasets are reported in Table 1. As it can be seen, for all scenarios, our proposed $LogSum + L_{2}$ procedure generally achieves higher or comparable classification performance than the $L_{1}$, $L_{EN}$ and $HLR$ methods. For example, in the Scenario 1 with $\rho$ = 0.6, our proposed method gained the 97.86% of accuracy, 95.38% of sensitivity and 100% of specificity, all of this data has increased by 6% for other methods. And whatever Scenario 1 or 2, the $LogSum + L_{2}$ methods always show the highest accuracy of training set, both $\rho$ = 0.2 and $\rho$ = 0.6. In summary, in the case of different scenarios and different values $\rho$, the *LogSum* + *L*_2_ penalized logistic regression model is always the best.

**Table 1:** Training results of different methods on the simulated datasets.

*ρ*	Method	Scenario							
		Accuracy		Sensitivity		Specificity		AUC	
		1	2	1	2	1	2	1	2
0.2	*L*_1_	90.00% (1.85%)	98.78% (0.37%)	91.30% (1.12%)	99.82% (0.01%)	88.73%(2.14%)	97.45% (0.49%)	97.12% (0.53%)	98.12% (0.32%)
	$L_{EN}$	87.14% (2.28%)	99.16% (0.12%)	86.96% (2.97%)	99.78% (0.03%)	87.32% (3.17%)	97.33% (0.52%)	95.08% (0.93%)	97.89% (0.35%)
	$HLR$	94.29% (0.59%)	98.65% (0.35%)	95.65% (0.62%)	99.82% (0.01%)	92.96% (1.26%)	98.31% (0.38%)	98.84% (0.21%)	98.53% (0.33%)
	$LogSum + L_{2}$	**100%** (0%)	**99.50%** (0.01%)	**100%** (0%)	**99.96%** (0.01%)	**100%** (0%)	**99.42%** (0.01%)	**100%** (0%)	**99.27%** (0.06%)
0.6	*L*_1_	91.43% (1.35%)	98.65% (0.26%)	87.69% (2.61%)	98.76% (0.13%)	94.67% (0.92%)	97.24% (0.29%)	97.37% (0.31%)	98.16% (0.28%)
	$L_{EN}$	85.71% (2.03%)	97.76% (0.31%)	69.23% (2.84%)	97.84% (0.20%)	100% (0%)	98.86% (0.22%)	96.04% (0.48%)	98.07% (0.34%)
	$HLR$	90.71% (1.76%)	98.65% (0.23%)	87.69% (1.48%)	99.12% (0.04%)	93.33% (0.81%)	98.21% (0.26%)	97.58% (0.40%)	98.54% (0.32%)
	$LogSum + L_{2}$	**97.86%** (0.21%)	**99.23%** (0.02%)	**95.38**% (0.62%)	**99.30%** (0.02%)	**100%** (0%)	**99.10%** (0.02%)	**100%** (0%)	**98.97%** (0.09%)

Numbers in parentheses are the standard deviations and the best results are highlighted in bold.

Table 2 shows test results of different methods on simulate datasets. We can find that the performance of the *LogSum* + *L*_2_ penalized logistic regression model is still the best one among the four methods. And in Scenario 1, whatever $\rho$ = 0.2 or $\rho$ = 0.6, the $LogSum + L_{2}$ approach shows similar values, but in Scenario 2, the sensitivity of the *LogSum* + *L*_2_ model is far apart, and its accuracy and specificity are not much different compared with other three methods.

**Table 2:** Test results of different methods on the simulated datasets.

*ρ*	Method	Scenario							
		Accuracy		Sensitivity		Specificity		AUC	
		1	2	1	2	1	2	1	2
0.2	$L_{1}$	75.00% (3.82%)	71.67% (3.19%)	78.31% (2.83%)	78.57% (2.88%)	74.19% (3.64%)	63.50% (4.31%)	86.76% (1.63%)	80.80% (2.37%)
	$L_{EN}$	78.33% (3.15%)	66.67% (4.18%)	79.54% (2.68%)	75.00% (3.07%)	87.10% (2.63%)	53.13% (6.16%)	84.43% (1.87%)	76.23% (3.49%)
	$HLR$	80.00% (1.86%)	65.00% (4.03%)	79.63% (2.66%)	71.43% (3.92%)	87.10% (2.52%)	58.76% (5.83%)	88.65% (1.31%)	77.23% (3.46%)
	$LogSum + L_{2}$	**85.00**% (1.55%)	**76.67**% (3.17%)	**86.21**% (1.43%)	**81.00**% (3.17%)	**89.87**% (1.96%)	**78.13**% (3.05%)	**93.99**% (0.62%)	**83.82**% (2.29%)
0.6	$L_{1}$	68.33% (4.01%)	58.33% (4.92%)	62.07% (4.65%)	59.09% (4.83%)	70.97% (3.62%)	57.89% (5.52%)	82.76% (1.94%)	65.67% (4.46%)
	$L_{EN}$	71.67% (3.61%)	56.67% (5.32%)	55.17% (5.18%)	63.64% (4.78%)	77.42% (2.95%)	44.74% (8.03%)	81.76% (2.43%)	59.93% (5.04%)
	$HLR$	73.33% (3.33%)	55.00% (5.57%)	58.62% (4.96%)	59.09% (5.02%)	80.65% (2.31%)	52.63% (5.24%)	86.76% (1.88%)	52.27% (5.71%)
	$LogSum + L_{2}$	**85.00**% (1.73%)	**70.00**% (2.83%)	**82.76**% (2.04%)	**69.09**% (3.11%)	**87.10**% (1.78%)	**76.32**% (2.71%)	**92.10**% (0.50%)	**71.77**% (4.32%)

Numbers in parentheses are the standard deviations and the best results are highlighted in bold.

Table 3 shows the feature selection of all competing regularization methods. As shown in Table 3, these are the *β*-Sensitivity and *β*-Specificity. The approximate results are similar to the previous two Tables. In the same $\rho$ value, the *LogSum* + *L*_2_ penalized logistic regression model contains the greatest number of features and highest sensitivity and specificity. And in different $\rho$ value, the performance of $\rho$ = 0.6 always greater than the performance of $\rho$ = 0.2.

**Table 3:** Results of $\beta$-sensitivity, $\beta$-specificity obtained by four methods. (Numbers in parentheses are the standard deviations and the best results are highlighted in bold).

*ρ*	Method	Scenario			
		*β*-Sensitivity		*β*-Specificity	
		1	2	1	2
0.2	$L_{1}$	73.45% (2.95%)	71.53% (2.94%)	99.90% (0.01%)	95.81% (0.52%)
	$L_{EN}$	73.16% (2.26%)	71.31% (2.32%)	99.95% (0.01%)	76.65% (2.73%)
	$HLR$	74.62% (3.05%)	73.15% (2.89%)	99.95% (0.01%)	95.45% (1.92%)
	$LogSum + L_{2}$	**82.57%** (2.58%)	**80.11%** (2.74%)	**99.95%** (0.01%)	**99.60%** (0.01%)
0.6	$L_{1}$	64.18% (3.56%)	62.43% (4.62%)	99.70% (0.01%)	95.00% (0.73%)
	$L_{EN}$	65.36% (3.63%)	63.34% (4.13%)	99.95% (0.01%)	76.00% (3.04%)
	$HLR$	65.41% (3.81%)	63.62% (4.51%)	99.90% (0.01%)	95.96% (0.65%)
	$LogSum + L_{2}$	**73.50%** (2.92%)	**72.31%** (3.86%)	**99.85%** (0.01%)	**99.24%** (0.01%)

### Analysis of real data

We use three publicly available lung cancer microarray datasets, which download from GEO (https://www.ncbi.nlm.nih.gov/geo/). Some detail information and introduction will be shown below:

1. GSE10072: Series GSE10072 is a gene expression signature of cigarette smoking and its role in lung adenocarcinoma development and survival. Tobacco smoking can cause 90% of lung cancer cases, but the changes in the level of the molecules that lead to cancer development and affect survival are still unclear.
2. GSE19188: Series GSE19188 is a dataset about gene expression for early stage Non-small-cell lung carcinoma (NSCLC). 156 tumors and normal samples are aggregated into the expected group. The prognostic characteristics of 17 genes showed the best correlation with the survival time after surgery.
3. GSE19804: Series GSE19804 is a dataset about Genome-wide screening of transcriptional modulation in non-smoking female lung cancer in Taiwan. Although smoking is a major risk factor for lung cancer, only 7% of women with lung cancer in Taiwan have a history of smoking, which is much lower than that of white women. Researchers extracted RNA from paired tumors and normal tissues for gene expression analysis to explain this phenomenon. This dataset and its reports comprehensively analyze the molecular characteristics of lung cancer in non-smoking women in Taiwan.

The GSE10072 dataset contains 22,284 microarray gene expression profiles and GSE19188 and GSE 19,804 both have 54,675 microarray gene expression profiles. As same as simulation data, we randomly divide the datasets such that 70% of the datasets become training samples and 30% become test samples. A brief introduction of these datasets is summarized in Table 4.

**Table 4:** Three publicly available lung cancer gene expression datasets.

Dataset	No. of probes	Classes (Class1/Class2)	No. of sample (Class1/Class2)
GSE10072	22,284	Normal/Lung Cancer	107 (49/58)
GSE19188	54,675	Normal/Lung Cancer	156 (88/91)
GSE19804	54,675	Normal/Lung Cancer	120 (60/60)

Table 5 describes the average training and test accuracies are obtained by different variable selection methods in the three datasets. It is easy to find that the performance of the *LogSum* + *L*_2_ penalized logistic regression model is better than other three approaches. For example, in terms of training accuracy, the $LogSum + L_{2}$ approach reached 99.43%, and other three methods are 98.32%, 99.04% and 98.21% respectively in GSE10072 dataset. In GSE19188 dataset, we observe the test accuracy of the $LogSum + L_{2}$ method is 75%, and other three methods are 51.46%, 47.56% and 46.19% respectively. From the number of selected genes, we can find the *LogSum* + *L*_2_ penalized logistic regression model always select the lowest number of genes and the $L_{EN}$ approach select the highest number of genes.

**Table 5:** Training and test accuracy and number of selected genes of three lung cancer datasets in four methods.

Data	Method	Training accuracy	Test accuracy	No. selected genes
GSE10072	$L_{1}$	98.32% (0.14%)	95.12% (0.31%)	23 (1.97)
	$HLR$	99.04% (0.04%)	98.4% (0.17%)	72 (8.45)
	*LEN*	98.21% (0.16%)	92.1% (0.94%)	11 (1.32)
	$LogSum + L_{2}$	**99.43%** (0.02%)	**99.15%** (0.08%)	7 (0.82)
GSE19188	$L_{1}$	97.11% (0.21%)	51.46% (6.05%)	72 (9.33)
	$HLR$	98.33% (0.09%)	47.56% (7.41%)	121 (10.34)
	*LEN*	96.3% (0.28%)	46.19% (5.23%)	17 (2.03)
	$LogSum + L_{2}$	**99.25%** (0.01%)	**75%** (3.44%)	10 (1.21)
GSE19804	$L_{1}$	99.05% (0.02%)	95.2% (0.61%)	37 (4.32)
	$HLR$	99.05% (0.02%)	94.6% (0.64%)	70 (7.73)
	*LEN*	97.14% (0.22%)	96.6% (0.58%)	9 (1.03)
	$LogSum + L_{2}$	**99.41%** (0.01%)	**98.45%** **(0.23%)**	6 (0.82)

Numbers in parentheses are the standard deviations and the best results are highlighted in bold.

**Figure 4:**
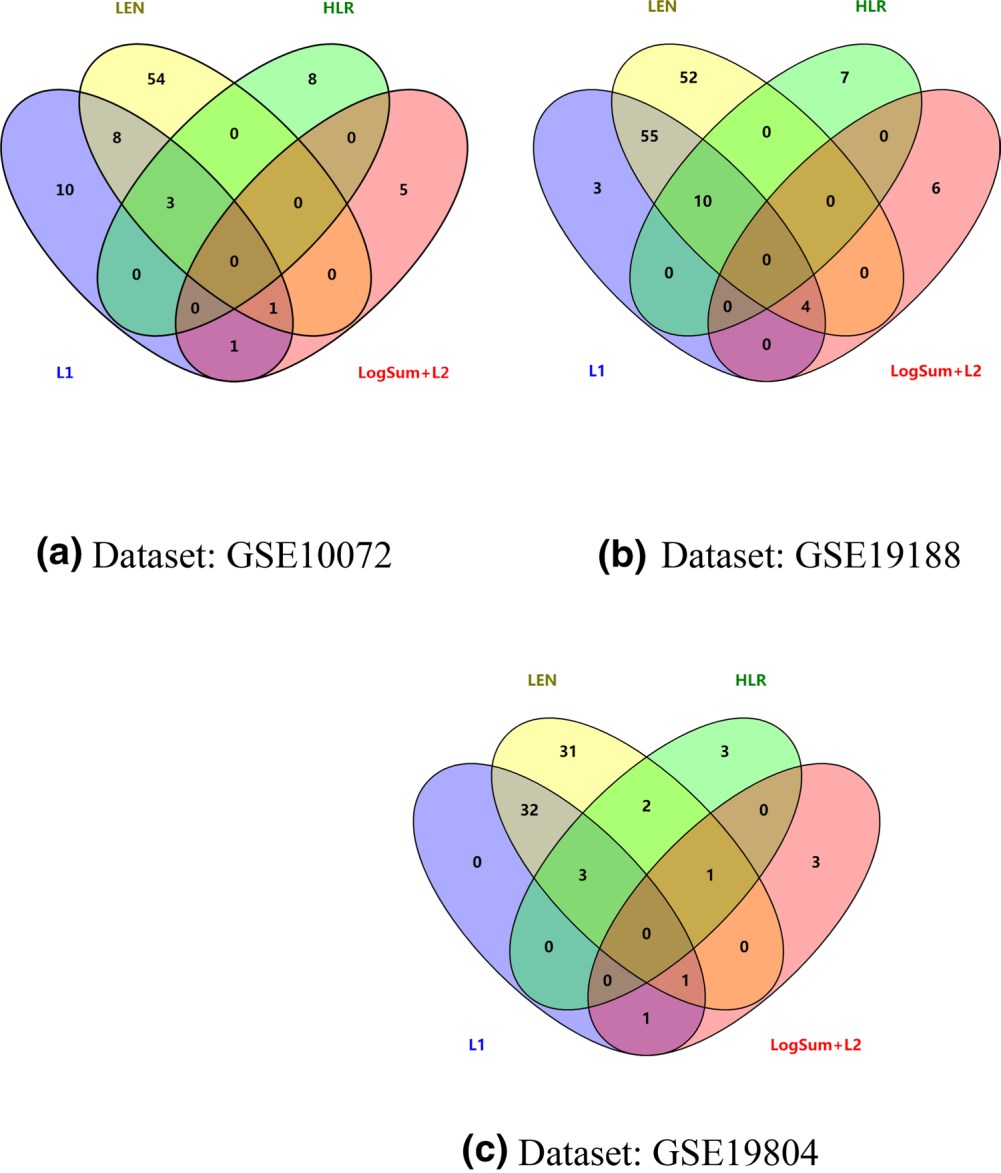
Venn diagram analysis of the results of $L_{1}$, $L_{EN}$, $HLR$ and $LogSum + L_{2}$ regularization methods.

In order to search the common gene signatures selected by the different methods, we used VENNY software to generate Venn diagrams. As show in Fig. 4, we consider the common gene signatures selected by the logistic regression model with $L_{1}$, $L_{EN}$, $HLR$ and $LogSum + L_{2}$ regularizations, which are the most relevant signatures of lung cancer. Many genes selected by the *LogSum* + *L*_2_ penalized logistic regression model do not appear in the results of the other three regularization methods. For example, the $LogSum + L_{2}$ approach selects 5, 6, and 3 unique genes from GSE10072, GSE19188 and GSE19804 datasets respectively. This means that the *LogSum* + *L*_2_ penalized logistic regression model can find the different genes and pathways related to lung cancer compared with other three regularization methods.

**Figure 5:**
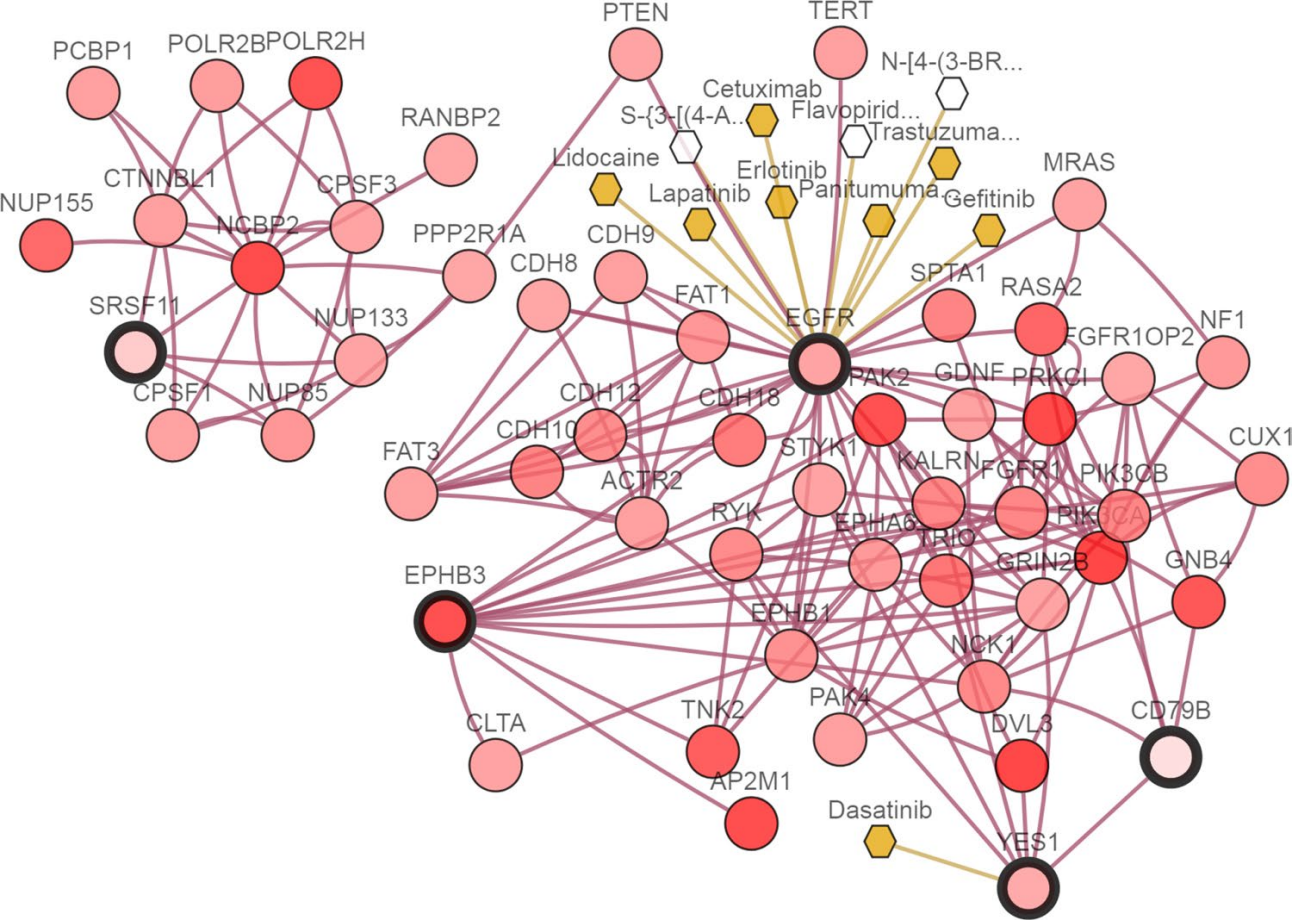
Maximum Integrative Network of features selected by the *LogSum* + *L*_2_ penalized logistic regression model in GSE10072 dataset.

**Figure 6:**
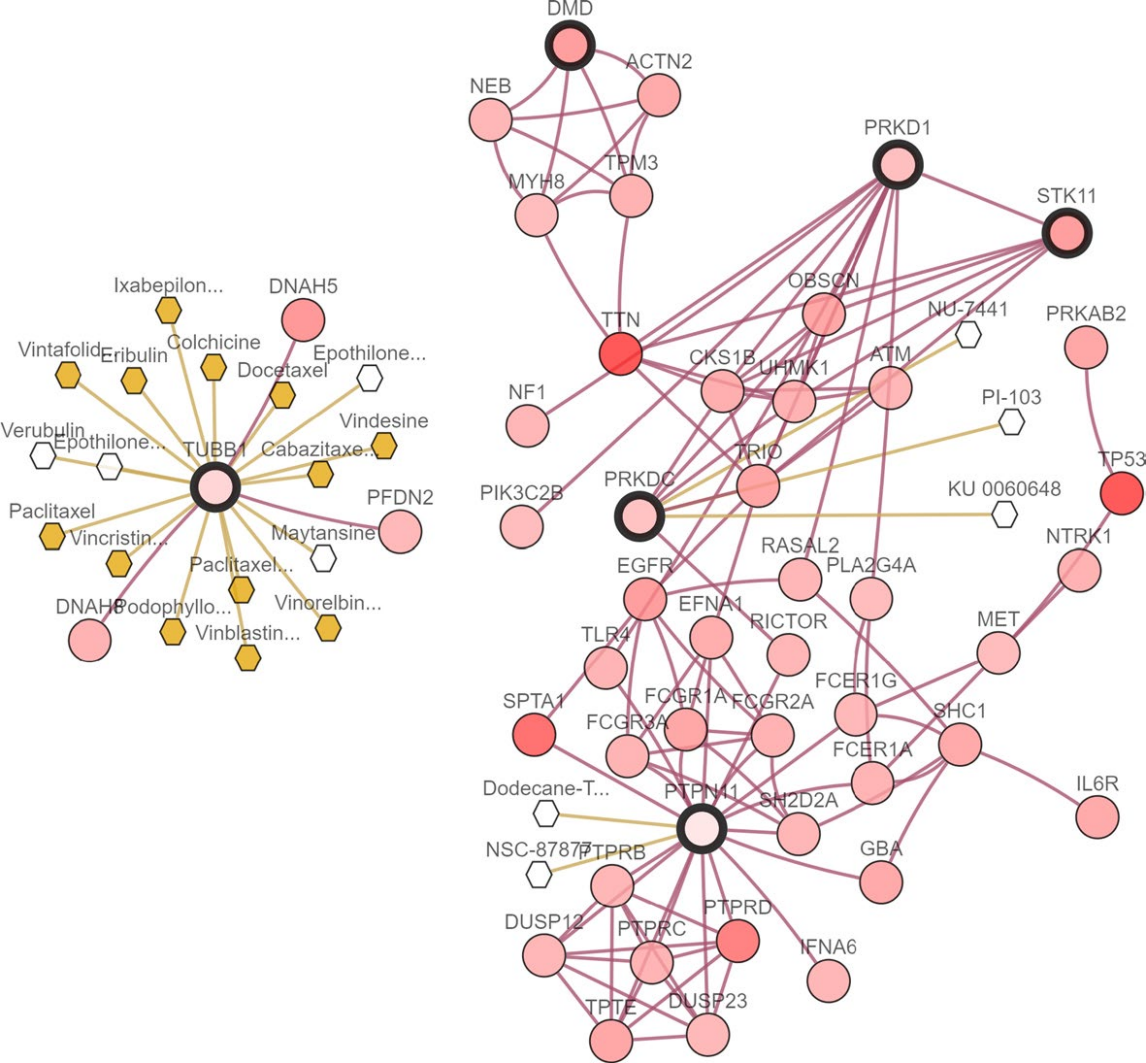
Maximum Integrative Network of features selected by the *LogSum* + *L*_2_ penalized logistic regression model in GSE19188 dataset.

**Figure 7:**
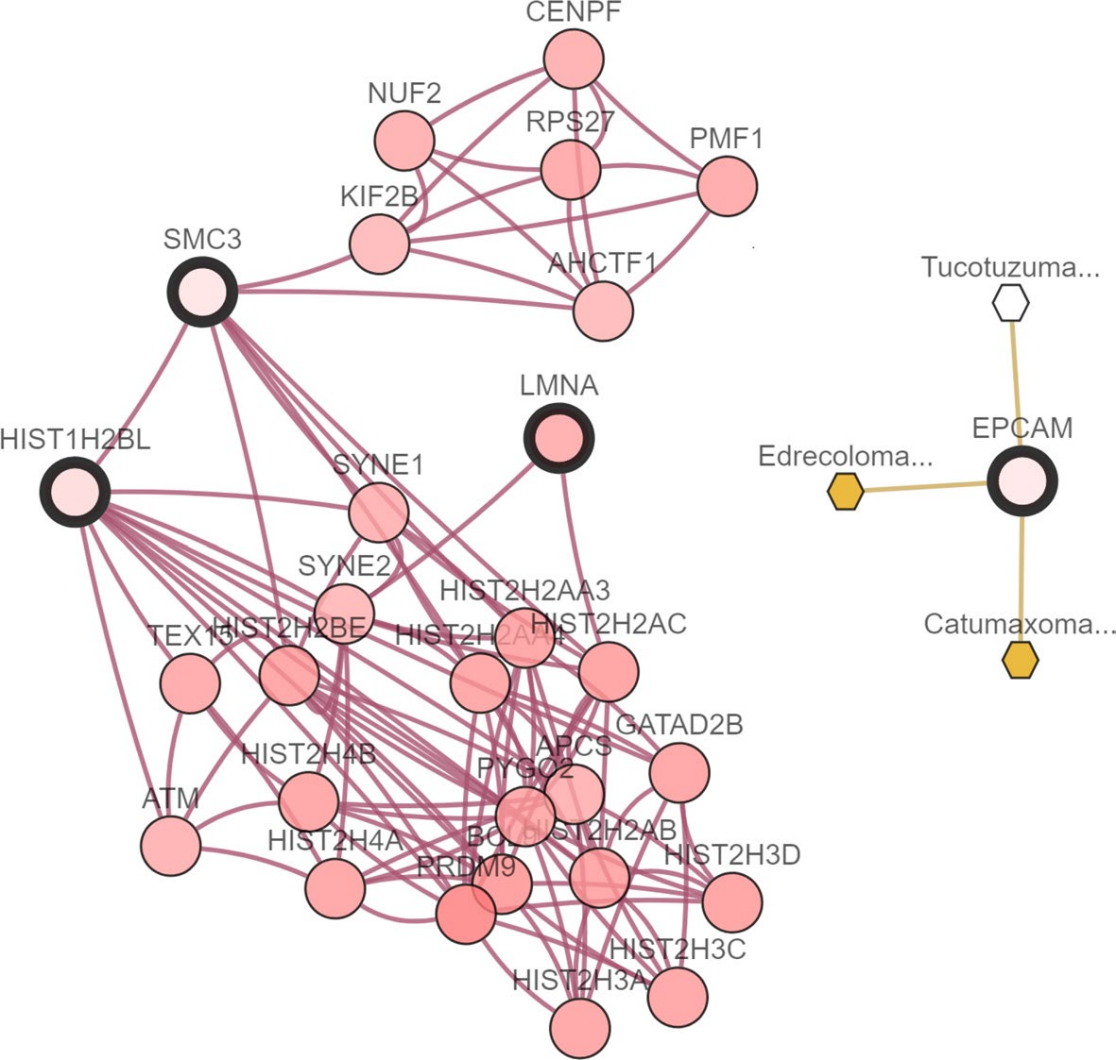
Maximum Integrative Network of features selected by the *LogSum* + *L*_2_ penalized logistic regression model in GSE19804 dataset.

Figures 5, 6 and 7 show the interactive networks of all the features selected by the *LogSum* + *L*_2_ penalized logistic regression model. The integrative networks among these selected features are represented by the cBioPortal from publicly lung cancer datasets. The circles with thick border represent the selected genes, and the rest circles with gradient color-coded represent genes according to their alteration frequencies in databases. The hexagons represent target drugs, and among of them some with yellow color represent the drugs approved by FDA. The links connected some selected genes represent that they have regulation correlations with group effect.

In GSE10072 dataset, from Fig. 5, we find a gene named EGFR, which has been conformed as the important target gene of NSCLC^40^. It belongs to ERBB receptor tyrosine kinase family, which include some other genes like HER2, HER3 and HER4. Due to observed patterns of oncogenic mutation of EGFR and HER2, many research works report their attractive option for targeted therapy in patients with NSCLC.

As shown in Fig. 6, three important genes TUBB1, PRKD1 and STK11 have been selected, and genes PRKD1 and STK11 have the regulation correlation with group effect from GSE19188 dataset. In fact, there are many drugs have been developed to target the gene TUBB1. And many research works report that genes PRKD1 and STK11 significantly influence the patients’ survival rates across all tumors^41^.

As shown in Fig. 7, four important genes EPCAM, SMC3, HIST1H2BL, and LMNA and their regulation correlations with group effect have been selected from GSE19804 dataset. Many research works report that the epithelial cell adhesion molecule (EPCAM) represents true oncogenes as the tumor-associated calcium signal transducer, and study the relationship between gene EPCAM and NSCLC^4,2^.

Table 6 summarizes that the genes were selected by the *LogSum* + *L*_2_ penalized logistic regression model. At the beginning of the experiments, the attribute of genes is prob set ID. Thus, we could transform prob set ID to gene symbol by using the website DAVID (https://david.ncifcrf.gov). According to the experimental results, the *LogSum* + *L*_2_ penalized logistic regression model can find some unique genes, which cannot be identified by other regularization models but are significantly related to the disease. Therefore, we believe that the *LogSum* + *L*_2_ penalized logistic regression model can accurately and efficiently identify cancer-related genes.

**Table 6:** The genes are selected by the *LogSum* + *L*_2_ penalized logistic regression model for different datasets.

Prob_ID	Gene symbol	Gene name
**Dataset: GSE10072**		
201839_s_at	EPCAM	Epithelial cell adhesion molecule (EPCAM)
200685_at	SRSF11	Serine and arginine rich splicing factor 11(SRSF11)
204600_at	EPHB3	EPH receptor B3(EPHB3)
205297_s_at	CD79B	CD79b molecule (CD79B)
202932_at	YES1	YES proto-oncogene 1, Src family tyrosine kinase (YES1)
201983_s_at	EGFR	Epidermal growth factor receptor (EGFR)
201596_x_at	KRT18	Keratin 18(KRT18)
**Dataset: GSE19188**		
204292_x_at	STK11	Serine/threonine kinase 11(STK11)
205880_at	PRKD1	Protein kinase D1(PRKD1)
208694_at	PRKDC	Protein kinase, DNA-activated, catalytic polypeptide (PRKDC)
205868_s_at	PTPN11	Protein tyrosine phosphatase, non-receptor type 11(PTPN11)
214250_at	NUMA1	Nuclear mitotic apparatus protein 1(NUMA1)
231657_s_at	CCDC74A	Coiled-coil domain containing 74A(CCDC74A)
220939_s_at	DPP8	Dipeptidyl peptidase 8(DPP8)
210704_at	FEZ2	Fasciculation and elongation protein zeta 2(FEZ2)
208601_s_at	TUBB1	Tubulin beta 1 class VI(TUBB1)
207660_at	DMD	Dystrophin (DMD)
**Dataset: GSE19804**		
1553655_at	CDC20B	Cell division cycle 20B(CDC20B)
201839_s_at	EPCAM	Epithelial cell adhesion molecule (EPCAM)
1552370_at	C4ORF33	Chromosome 4 open reading frame 33(C4orf33)
1556925_at	SMC3	Structural maintenance of chromosomes 3(SMC3)
207611_at	HIST1H2BL	Histone cluster 1 H2B family member l(HIST1H2BL)
1554600_s_at	LMNA	Lamin A/C(LMNA)

## Discussion and conclusion

Successful identification of gene biomarkers and biological pathways can significantly improve the accuracy of diagnosis and help machine learning models have better performance on classification of different types of cancer. Many researchers used the logistic regressions with optimization methods for binary cancer classification. However, the traditional logistic regression model has two obvious shortcomings: feature selection and overfitting problems. In this paper, we proposed the $LogSum + L_{2}$ penalized logistic regression model. Our proposed method can not only select sparse features (biomakers), but also identify the groups of the relevant features (gene pathways). The coordinate decent algorithm is used to solve the *LogSum* + *L*_2_ penalized logistic regression model. We also evaluate the capability of our proposed method and compare its performance with other regularization methods. The results of simulations and real experiments indicate that the proposed method is highly competitive among several state-of-the-art methods. The disadvantage of the proposed method is its three regularization parameters need to be tuned by the *k*-fold cross-validation approach.

In recent years, increasing associations between of microRNAs (miRNAs) and human diseases have been identified. Based on accumulating biological data, many computational models for potential miRNA-disease associations inference have been developed^43–46^. We will apply the proposed *LogSum* + *L*_2_ penalized logistic regression model to identify the non-coding RNA biomarker of human complex diseases as the future direction of our research.
